# Pathological Museum of the Medical Institute, L.

**Published:** 1840

**Authors:** 


### PATHOLOGICAL MUSEUM.

The undersigned, professor of Pathological Anatomy in the *Louisville Medical Institute*, being engaged in founding a cabinet of morbid specimens, respectfully solicits contributions to it from his brethren in the west. They may be sent in alcohol, or a solution of corrosive sublimate, or even in proof spirits, when they are not of a kind to be dried. In all cases, they ought to be accompanied with notes of the disease by which the patient or the animal was destroyed. When placed in the Museum for permanent preservation and exhibition, they will be labelled with the names of the donors. The extensive water communication which the city of Louisville maintains with all parts of the west and south, is highly favorable to the transmission of specimens, which may always be done, at the expense of the Institute. D.

